# Titanium Dioxide Nanoparticles Altered the lncRNA Expression Profile in Human Lung Cells

**DOI:** 10.3390/ijerph20021059

**Published:** 2023-01-06

**Authors:** Ying Ma, Jiaqi Shi, Yi Zhang, Zhangjian Chen, Guang Jia

**Affiliations:** 1 Department of Occupational and Environmental Health Sciences, School of Public Health, Peking University, Beijing 100191, China; 2 Beijing Key Laboratory of Toxicological Research and Risk Assessment for Food Safety, School of Public Health, Peking University, Beijing 100191, China

**Keywords:** titanium dioxide nanoparticles, respiratory toxicity, BEAS-2B cells, lncRNA, epigenetics

## Abstract

Respiration is considered to be the main occupational or environmental exposure pathway of titanium dioxide nanoparticles (TiO_2_ NPs), and the lung is considered to be the target organ of respiratory exposure; however, the mechanism of respiratory toxicity is not fully understood. In this study, the effect of TiO_2_ NPs on the expression profile of long non-coding RNA (lncRNA) in bronchial epithelial cells (BEAS-2B) was investigated to understand their potential toxic mechanism. BEAS-2B cells were treated with 100 μg/mL TiO_2_ NPs for 48 h, then RNA sequencing was performed to screen the differential lncRNAs compared with the control group, and the enrichment pathways of the differentially expressed lncRNAs were further analyzed using the Kyoto Encyclopedia of Genes and Genomes (KEGG). The results identified a total of 45,769 lncRNAs, and 277 different lncRNAs were screened. KEGG pathway analysis showed that the targeted mRNAs of these different lncRNAs were enriched in the pyrimidine metabolism pathway. This work demonstrates that TiO_2_ NPs could alter the lncRNA expression profile in BEAS-2B cells, and epigenetics may play a role in the mechanism of respiratory toxicity induced by TiO_2_ NPs.

## 1. Introduction

Nanomaterials are now widely employed in a variety of industries, including clothing, food, housing, and transportation, thanks to the ongoing development of nanotechnology and its applications. One of the most popular nanomaterials, titanium dioxide nanoparticles (TiO_2_ NPs), have three different crystal structures: rutile, anatase, and plate titanium. They are widely utilized in the cosmetics, medicinal, and food industries due to their excellent whiteness, opacity, good antibacterial properties, high heat stability, hydrophilicity, and chemical stability. In occupational and personal settings, inhalation of TiO_2_ NPs is regarded as the main point of human-body entrance. In the epidemiological survey conducted by Zhen et al., it was found that seven workers exposed to 0.319 to 6.258 mg/m^3^ showed pulmonary dysfunction with decreased maximum spontaneous ventilation and expiratory flow [1]. In Yamano’s [2] study, the incidence of bronchoalveolar adenomas was significantly increased in female mice after 26 weeks of inhalation of 32 mg/m^3^ TiO_2_ NPs. The deterioration of building materials in a living environment also poses a concern for the release of titanium dioxide into the atmosphere [3]. For instance, free TiO_2_ NPs were released from the surface of ceramic tiles [4], some free TiO_2_ NPs were emitted from aged paint [5], and commercial photocatalytic nanocoatings of building materials can release free TiO_2_ NPs into the air during weathering, use, or maintenance [6]. However, the key mechanisms of TiO_2_ NPs-induced respiratory toxicity are not fully understood and require further investigation.

High-throughput sequencing results indicate that the human genome has been extensively transcribed. To date, 127,802 long non-coding RNA (lncRNA) transcripts have been identified and examined (www.LNCipedia.org, accessed on 13 November 2022). lncRNA is similar to messenger RNA (mRNA) in that both have a 5′ 7-methylguanosine cap and a 3′ poly(A) tail. However, lncRNAs lack coding ability. Although not translated into proteins, they remain a functional molecule which interacts with other coding and non-coding RNAs, DNAs, and proteins in cells to participate in many cellular processes [7,8]. Previous studies have shown that lncRNAs are abnormally expressed under microenvironmental stress caused by heavy nanomaterials (titanium dioxide, silver, and gold) [9]. Therefore, lncRNA expression has attracted attention as a new target for epigenetic toxicology [10].

In this study, the effects of titanium dioxide exposure on lncRNA expression profiles in human lung cells (BEAS-2B) were assessed through RNA sequencing, offering fresh insights into the epigenetic impacts of TiO_2_ NPs.

## 2. Materials and Methods

### 2.1. Physicochemical Properties of TiO_2_ NPs and Cell Culture

TiO_2_ NPs in this study were purchased from Shanghai Macklin Biochemical Co., Ltd. (Shanghai, China). The physicochemical properties of TiO_2_ NPs were described in our previous articles [11]. Human normal bronchial epithelial cells (BEAS-2B), purchased from the American Type Culture Collection (ATCC, Rockville, MD, USA), were used in this study. Cells were cultured in Dulbecco’s modified Eagle medium (DMEM, HyClone, Logan, UT, USA) with 2% 200 mM glutamine (Lonza, Basel, Switzerland) and 10% FBS (Gibco, Gaithersburg, MD, USA).

### 2.2. Cell Experimental Design

TiO_2_ NPs (100 μg/mL) were dispersed in phosphate buffered solution (PBS, HyClone, Logan, UT, USA) and sonicated for 15 min. Then, BEAS-2B cells were incubated with the suspensions of TiO_2_ NPs for 48 h.

### 2.3. Construction of cDNA Libraries and RNA Sequencing

An miRNA extraction kit (Cat TR205-200, Tanmo, Beijing, China) was used to extract total RNA. Qualified total RNA was further purified by an RNAClean XP Kit (Cat A63987, Beckman Coulter, Inc., Kraemer Boulevard Brea, CA, USA) and RNase-Free DNase Set (Cat 79254, QIAGEN, Hilden, Germany). A VAHTS Ribo-off rRNA Depletion Kit (Cat N406-02, Vazyme, Nanjing, China), VAHTS Universal V6 RNA-seq Library Prep Kit for Illumina^®^ (Cat NR604-02, Vazyme, Nanjing, China), VAHTS DNA Clean Beads (Cat N411-03, Vazyme), Qubit™ dsDNA HS Assay Kit (Cat Q32854, Invitrogen, Waltham, MA, USA), and Agilent High Sensitivity DNA Kit (Cat 5607-4626, Agilent) were used to remove rRNA from the purified total RNA and then fragment mRNA. After first-strand cDNA synthesis was produced utilizing random primer reverse transcription, and second-strand cDNA synthesis. After going through end repair, the produced cDNA was 3′ adenylated. These 3′ adenylated cDNA fragments’ ends were joined by adapters. To enrich the cDNA fragments, many rounds of PCR amplification using PCR Primer Cocktail and PCR Master Mix were carried out. The cDNA was then sequenced with a high-throughput sequencer (Illumina HiSeq 2000/2500, San Diego, CA, USA).

### 2.4. Analysis of lncRNA

Gffcompare (version: 0.9.8) was applied to compare the transcript information obtained by concatenating StringTie (version: 1.3.0) with the transcript information of known reference genomes and extract three types of transcripts. When the open reading frame (ORF) was less than 300 bp, the number of exons was larger than or equal to 2, and the transcription length was greater than or equal to 200 bp, lncRNAs were screened. The protein families database (PFAM), coding potential calculator (CPC), and coding on coding index (CNCI) were used to filter out lncRNAs with coding potential and obtain the predicted lncRNA sequences. Specifically, transcripts with CPC score < 0, CNCI score < 0 and insignificant PFAM alignment were selected as potential lncRNAs.

lncRNA expression was quantified with StringTie (version: 1.3.0). The fragments per kilobase of transcript per million fragments mapped (FPKM) value was used as an indicator to measure the expression level of lncRNAs. lncRNA analysis of differences between samples was carried out by edgeR, multiple hypothesis testing was carried out after obtaining the *p* value, and the p-value threshold was determined by controlling the false discovery rate (FDR). The q value was adjusted by p value. Meanwhile, we calculated the differential expression multiple according to the FPKM value, namely, fold change. A q value ≤ 0.05 and the fold change ≥ 2 was considered to be a differential lncRNA.

The structures of lncRNAs and mRNAs were compared and analyzed to observe the differences in transcription length, exon number and expression level between lncRNAs and mRNAs. Trans and cis regulation both anticipated the target genes. Trans prediction was performed using a database of mRNAs of the species. Sequences with complementarity or similarity in sequence were first selected using blast, and then the complementary energy between the two sequences was calculated using RNAplex to select sequences above a threshold. The gene with a distance of less than 10 kb from the lncRNA was selected as the target gene for cis action. The target genes of differential lncRNAs and differential mRNAs were intersected, and KEGG was used to calculate the number of differential lncRNAs in each pathway.

### 2.5. Statistical Analysis

The numerical statistics were presented as the mean ± standard deviation (m ± SD) of at least three measurements. Orthogonal partial least-squares discriminant analysis (OPLS-DA) of the transcribed data was performed via the Metaboanalyst 5.0 website (https://www.metaboanalyst.ca/ (accessed on 7 November 2022)). To view the data distribution of each group, the first and second principal-component scores were displayed as OPLS-DA scores. The threshold was set as q value ≤ 0.05 following the correction of the calculated *p* value by multiple hypothesis testing, which was defined as the KEGG pathway significantly enriched in differential lncRNAs, and the top 30 KEGG pathways with enrichment factors were displayed.

## 3. Results

### 3.1. Characterization of the TiO_2_ NPs

Here, the physicochemical properties of TiO_2_ NPs were briefly described (Table 1). Transmission electron microscopy (TEM) showed that the primary particle size of titanium dioxide was 25.12 ± 5.64 nm. The particle size of its hydration in different solutions varies, and the hydrated sizes in ultrapure water and DMEM were 609.43 ± 60.35 nm and 878.93 ± 105.75 nm, respectively. The zeta potential in the two solutions was also different, −8.33 ± 0.22 mV and −15.20 ± 0.92 mV, respectively, suggesting that the stability of TiO_2_ NPs varies somewhat depending on the solution.

### 3.2. Predictions and Annotations of lncRNAs

**Figure 1:**
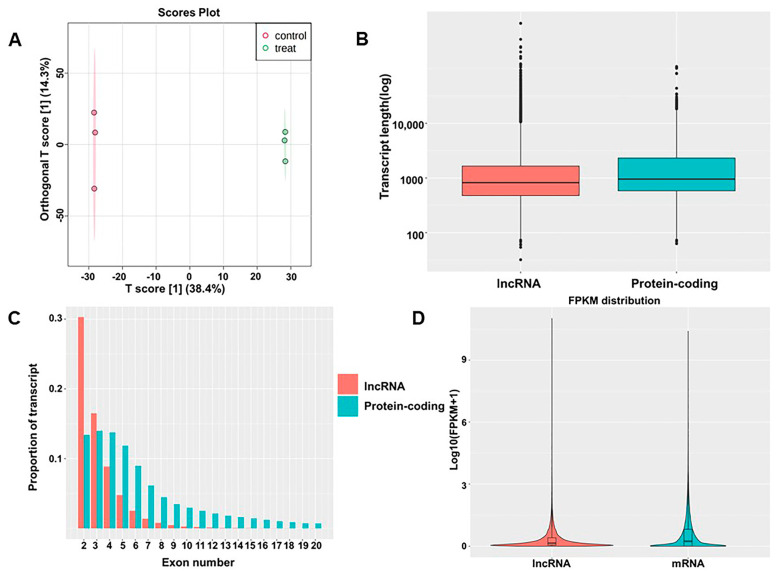
Identification of lncRNAs and comparison with mRNAs. (**A**) OPLS−DA of lncRNAs; (**B**) lengths of lncRNAs and mRNAs; (**C**) exon numbers of lncRNAs and mRNAs; (**D**) expression levels of mRNAs and lncRNAs.

As mentioned in previous articles [11], after 48 h of culture with TiO_2_ NPs, BEAS-2B cell viability was 71.5%. To determine whether TiO_2_ NPs exposure induces changes in lncRNA expression, total RNA from BEAS-2B cells exposed to TiO_2_ NPs was compared to the control. A total of 45,769 lncRNAs were detected, including 45,548 known lncRNAs and 221 predicted lncRNAs. These transcripts served as the basis for OPLS-DA analysis, which distinguished the TiO_2_ NPs-exposed group from the control group (Figure 1A). The box plot demonstrates the length of lncRNAs and mRNAs, indicating that lncRNAs were shorter than mRNAs (Figure 1B). Approximately 30% of lncRNAs were composed of two exons, while the remaining lncRNAs with multiple exons accounted for a relatively low proportion (Figure 1C). However, compared to lncRNAs, the number of exons in mRNAs was significantly higher. Expression-level analysis showed that the overall expression level of lncRNAs was slightly lower than the expression level of mRNAs (Figure 1D). lncRNAs had fewer exons, a shorter sequence length, and a lower expression level than mRNAs, indicating that the two have distinct sequence components.

### 3.3. Differential Expression of lncRNAs

**Figure 2:**
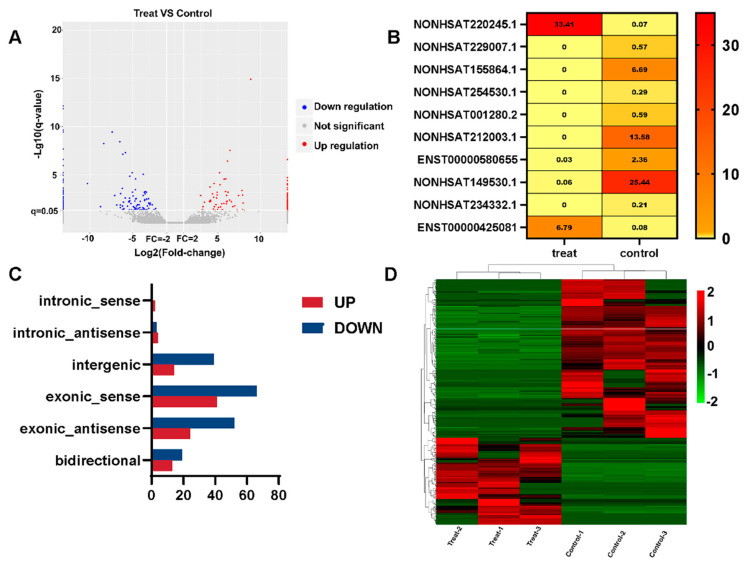
lncRNA differential expression analysis of TiO_2_ NPs. (**A**) The number of upregulated and downregulated genes in the treatment group is shown on a volcano map of differentially expressed genes; (**B**) the top 10 lncRNAs with the lowest q value in the treatment group and the control group are displayed in the heatmap, and the FPKM value of each lncRNA is displayed. The FPKM value is represented by different colors, with yellow to red values increasing; (**C**) the relative expression of differential lncRNAs is shown in a histogram. The downregulated genes are shown in blue, while the upregulated genes are represented in red; (**D**) the distinctive differences between the treatment group and control group are shown via a heatmap of cluster analysis. The downregulated genes are shown in green, while the upregulated genes are represented in red.

In order to identify differentially expressed (DE) lncRNA transcripts with possible biological significance, this work employed a two-fold change and q value ≤ 0.05. The total number of DE lncRNA transcripts across all exposures was reduced to 277 as a result, with 179 lncRNAs being downregulated and 98 lncRNAs being upregulated (Figure 2A). The q values were sorted in ascending order. Below is a heatmap of the top 10 lncRNAs with the lowest q values (Figure 2B). The TiO_2_ NPs treatment group (FPKM) had the greatest NONHSAT220245.1 expression level, whereas the control group had the highest NONHSAT149530.1 expression level (FPKM). The NONCODE database shows that NONHSAT220245.1 and NONHSAT149530.1 are significantly expressed in the brain and skeletal muscle, respectively, according to the expression profile of lncRNAs in human tissues. Among the differentially upregulated lncRNAs, there were 2 intronic_sense, 4 intronic_antisense, 14 intergenic, 41 exonic_sense, 24 exonic_antisense, and 13 bidirectional lncRNAs. In downregulated lncRNAs, there was 0 belonging to intronic_sense, 3 intronic_antisense, 39 intergenic, 66 exonic_sense, 52 exonic_antisense, and 19 bidirectional (Figure 2C). A hierarchical cluster based on the relative expression of these 277 lncRNAs revealed TiO_2_ NPs-dependent alterations in the transcriptome of BEAS-2B cells (Figure 2D).

### 3.4. Enrichment Analysis of DE lncRNA Target Genes

**Figure 3:**
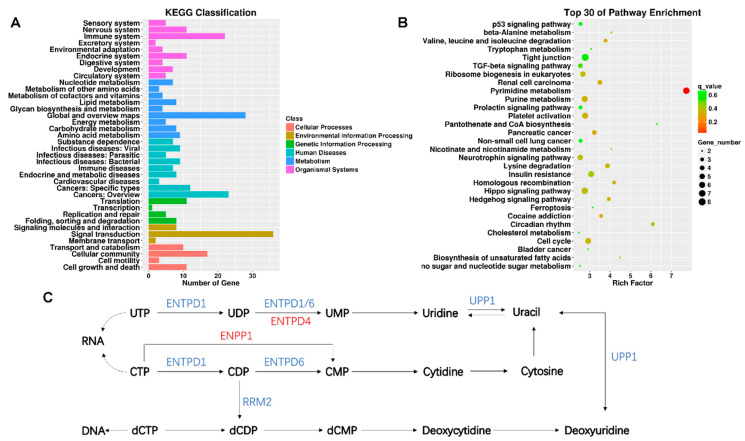
KEGG analysis of differentially expressed lncRNAs target genes. (**A**) The number of genes in different KEGG classifications is shown; (**B**) the results of KEGG pathway enrichment analysis of differentially expressed genes are shown in scatter plots. Where Rich Factor = (the number of differential genes in a KEGG term/the number of differential genes in the KEGG database)/(the number of genes in a KEGG term/the number of genes in the KEGG database). The degree of enrichment increases with the rich factor. The more significant the enrichment, the smaller the value of the q value, which is the p value after multiple hypothesis testing has been corrected. In the Rich Factor, only the top 30 KEGG entries are displayed; (**C**) showing altered pathways in pyrimidine metabolism, blue is the gene with decreased expression compared to the control group, and red is the gene with increased expression compared to the control group.

KEGG enrichment analysis was performed for intersecting genes. The KEGG classification with the most gene-number changes was signal transformation, which belongs to environmental information processing (Figure 3A). According to the rich factor and the q value (FDR adjusted p value), the pyrimidine metabolism pathway was the most significant pathway of enrichment (Figure 3B). KEGG classification and pathway enrichment maps of up- and down-regulated lncRNAs target genes were shown in Figure S1. There are several genes in the pyrimidine metabolic pathway that underwent significant changes, including ectonucleotide pyrophosphatase/phosphodiesterase 1 (ENPP1), ribonucleotide reductase regulatory subunit M2 (RRM2), ectonucleoside triphosphate diphosphohydrolase 4 (ENTPD4), ectonucleoside triphosphate diphosphohydrolase 6 (ENTPD6), uridine phosphorylase 1 (UPP1), and ectonucleoside triphosphate diphosphohydrolase 1 (ENTPD1) (Figure 3C). Next, the target genes of the DE lncRNAs were intersected with those of the DE mRNAs (Table 2). As a result, among the 277 screened DE lncRNAs, 23 differentially expressed lncRNA-matched mRNAs were found to have undergone significant changes. For example, genes associated with respiratory diseases and cell proliferation (CA5B, ID2, CARD14 and TRIM29) were significantly altered.

## 4. Discussion

The fate of inhaled TiO_2_ NPs has been studied in the lung, but little is known about the changes in lncRNAs in lung cells. Previous studies have revealed that inhalation of TiO_2_ NPs can alter epigenetic modifiers, which control histone deacetylation in lung cells [12]. The constant increase in TiO_2_ led to the presence of airborne TiO_2_ NPs. In addition to potential high exposures in the workplace, chronic low-dose exposures may occur in the general population. It is, therefore, important to assess the health impacts of such exposure, especially on lung cells. This work revealed alterations in the lncRNA expression profile in BEAS-2B cells after exposure to TiO_2_ NPs. The study found that after exposure to 100 µg/mL TiO_2_ NPs for 48 h, the expression profile of lncRNAs changed, and the pyrimidine metabolism pathway may be crucial in the development of TiO_2_ NPs-induced lung cytotoxicity. In this work, epigenetic modifications in BEAS-2B cells were initially revealed by high-throughput sequencing, and the respiratory toxicity of TiO_2_ NPs was evaluated by bioinformatics analysis.

The National Institute for Occupational Safety and Health classifies inhaled ultrafine titanium dioxide as a possible occupational carcinogen (https://www.cdc.gov/niosh/index.htm (accessed on 7 November 2022)). TiO_2_ NPs-induced respiration toxicity has been widely studied. In-vivo studies have shown an increased incidence of bronchoalveolar adenomas and restricted airflow in the airway following TiO_2_ NPs inhalation [13,14]. TiO_2_ NPs were discovered to cause DNA damage in in-vitro studies utilizing the same cells as the current study [15,16]. A total of 277 differential lncRNAs were found in this study, suggesting epigenetic changes. Epigenetic alterations are heritable modifications which do not include DNA sequence changes. Recent in-vitro investigations into TiO_2_ NPs have demonstrated that, in addition to cytotoxicity and genotoxicity, exposure to TiO_2_ NPs causes epigenetic alterations in cells [17]. A significant epigenetic mechanism known as DNA methylation includes the insertion of methyl groups into CpG sites [18]. CpG site methylation or demethylation is critical for sustaining cell-specific gene expression. Incubation of A549 cells with anatase TiO_2_ NPs for 24 h induced poly (ADP-ribose) polymerase 1 (PARP-1) hypermethylation and crosstalk with reactive oxygen species [19]. TiO_2_ NPs can cause genotoxicity in lung cells by causing DNA to repair gene promoter methylation in BEAS-2B and A549 cells [20]. Therefore, it is crucial to further study epigenetic mechanisms, such as changes in lncRNAs in this study, for the assessment of TiO_2_ NPs exposure risk.

lncRNAs, which function as epigenetic modification factors by controlling gene expression, are significant participants in the epigenetic mechanism [21]. lncRNAs participate in the epigenetic regulatory processes of X chromosome inactivation and genomic imprinting [22]. By ‘coating’ the surface of the inactivated X chromosome, lncRNA silences genes in cis [23]. However, their defects or abnormal expression are associated with the disease process [24]. In a study of non-small-cell lung cancer, upregulation of SBF2-AS1 and AGAP2-AS1 promoted tumor-cell proliferation by negatively regulating p21, KLF2 and LATS2 [25,26]. TUG1 triggers lung-cell dysfunction after particulate matter exposure by increasing CUGBP elav-like family member 1 (CELF1) and p53 expression [27]. One of the genes significantly altered in this study, CA5B, is a class of genes mainly distributed in mitochondria. In Konwar’s study, CA5B was shown to be associated with respiratory diseases [28]. Numerous biological processes, including cell proliferation, senescence, differentiation, apoptosis, angiogenesis, and tumor transformation, have been linked to the ID2 gene [29,30]. Lung adenocarcinoma cells’ motility, invasion, proliferation, and colony formation can be inhibited by the overexpression of ID2, although this effect can be reversed by silencing ID2 [31]. CARD14 encodes a protein containing a caspase recruitment domain, which is involved in cell adherence, signal transduction and other cellular processes. Knockdown of CARD14 can prevent breast cancer cells from proliferating and migrating, stop the cell cycle at the G1/S juncture, and increase cell death [32]. Another overexpressed gene in our study, TRIM29, is also associated with tumor cell proliferation and migration. The miR- 761/TRIM29/PHLPP1 axis is activated by circL4R to facilitate the proliferation and spread of colorectal cancer cells [33]. By controlling the expression of their target genes, differentially expressed lncRNAs change the internal physiology of cells, which leads to cytotoxicity.

The biomolecules pyrimidines are essential. They make up the structural components of pterins, folates, vitamins, nucleotides, and nucleic acids, all of which play important functions in the cell. Pyrimidine metabolism is significantly involved in procedures including RNA and DNA synthesis, the creation of UDP sugars for the glycosylation of proteins and lipids, and the creation of CDP-activated membrane phospholipid precursors [34]. In Poirier’s study [35], inhibition of UMP synthase promoted the synthesis of triacylglycerol and the formation of cytoplasmic lipid droplets. In this study, through KEGG enrichment analysis, it was found that TiO_2_ NPs induced significant changes in ENPP1, RRM2, ENTPD4, ENTPD6, UPP1 and ENTPD1 in the pyrimidine metabolic pathway in BEAS-2B cells, and they mainly regulated the synthesis of uracil and deoxycytidine. Uracil is one of the most common noncanonical bases in DNA and is produced through spontaneous and enzymatic cytosine deamination and results in U:G mispairs [36]. The decreased incorporation of uracil into nucleotides and nucleic acids was observed during programmed cell death [37]. Numerous studies have discovered that pyrimidine metabolism is associated to respiratory disease with the advancement of contemporary non-targeted histology techniques. Wang et al. discovered that controlling the metabolism of pyrimidines effectively treated asthma [38]. Differential metabolites were discovered to be enriched in the pyrimidine metabolic pathway in a study that used untargeted lipidomics to analyze the impact of coal-dust exposure on blood metabolites [39].

The main advantage of this article is the combination of high-throughput sequencing and bioinformatics to analyze the alteration of lncRNAs and the involved pathways. However, this is merely a preliminary investigation on epigenetics, and the DE lncRNAs and mRNAs discovered lacked substantial verification. Future research should focus further on the reaction of the pyrimidine metabolism pathway to exposure to TiO_2_ NPs and associated.

## 5. Conclusions

In conclusion, this study illustrated the effect of TiO_2_ NPs exposure on the lncRNA expression profile of BEAS-2B cells. According to the OPLS-DA and cluster analysis of lncRNAs in different treatments, the biological correlation between the lncRNA expression profile and TiO_2_ NPs lung cytotoxicity was proven. The target genes of the differentially expressed lncRNAs intersected with those of mRNAs, which are involved in cell migration and proliferation, offering fresh insights into cellular processes following exposure. Additionally, given that KEGG enriched pyrimidine metabolic pathways, we recommend that future research be carried out to better examine uracil and deoxycytidine as well as the associated epigenetic processes. This work broadens our understanding of how mRNAs and lncRNAs interact as well as the methodology for evaluating the pulmonary toxicity of TiO_2_ NPs, which is crucial for a thorough evaluation.

## Figures and Tables

**Table 1:** Physicochemical properties of TiO_2_ NPs.

Type	Primary Size (nm)	Hydrated Sizes in Ultrapure Water (nm)	Hydrated Sizes in DMEM (nm)	Zeta Potential in Ultrapure Water (mV)	Zeta Potential in DMEM (mV)
Anatase	25.12 ± 5.64	609.43 ± 60.35	878.93 ± 105.75	−8.33 ± 0.22	−15.20 ± 0.92

**Table 2:** The target genes of the differentially expressed lncRNAs intersected with those of the mRNAs.

LncRNA_ID	Treat ^a^	Control ^a^	q-Value	Gene_ID	Gene_Name	Treat ^a^	Control ^a^	q-Value
**NONHSAT248795.1**	0.00	0.33	3.31 × 10^−2^	**ENSG00000218336**	**TENM3**	0.55	2.03	1.25 × 10^−2^
**NONHSAT226241.1**	0.00	0.13	3.31 × 10^−2^	**ENSG00000187634**	**SAMD11**	0.03	1.05	1.59 × 10^−5^
**MSTRG.48669.1**	0.37	1.81	3.31 × 10^−2^	**ENSG00000137573**	**SULF1**	0.19	0.98	2.29 × 10^−3^
**NONHSAT169524.1**	2.89	0.04	1.45 × 10^−3^	**ENSG00000165555**	**NOXRED1**	0.07	0.22	3.31 × 10^−2^
**NONHSAT080219.2**	0.04	2.50	8.61 × 10^−3^	**ENSG00000158445**	**KCNB1**	0.32	2.59	1.49 × 10^−9^
**NONHSAT193964.1**	0.00	1.47	6.65 × 10^−4^	**ENSG00000241472**	**PTPRG-AS1**	0.11	0.30	1.08 × 10^−5^
**NONHSAT056352.2**	0.35	0.04	4.25 × 10^−2^	**ENSG00000141527**	**CARD14**	0.02	0.05	1.18 × 10^−4^
**ENST00000315302**	0.01	0.65	4.50 × 10^−3^	**ENSG00000218336**	**TENM3**	0.55	2.03	3.72 × 10^−2^
**ENST00000572730**	0.00	1.07	1.11 × 10^−6^	**ENSG00000141527**	**CARD14**	0.02	0.05	5.62 × 10^−20^
**NONHSAT160825.1**	0.13	1.90	2.26 × 10^−2^	**ENSG00000137699**	**TRIM29**	0.10	0.43	3.59 × 10^−28^
**NONHSAT226246.1**	0.01	0.47	3.31 × 10^−2^	**ENSG00000187634**	**SAMD11**	0.03	1.05	1.43 × 10^−15^
**NONHSAT162857.1**	0.00	0.22	5.65 × 10^−3^	**ENSG00000152137**	**HSPB8**	0.16	1.54	1.61 × 10^−2^
**NONHSAT257137.1**	0.00	0.27	5.03 × 10^−8^	**ENSG00000204054**	**LINC00963**	0.68	1.93	4.77 × 10^−22^
**NONHSAT069007.2**	0.13	0.00	1.00 × 10^−2^	**ENSG00000115738**	**ID2**	2.50	8.27	8.26 × 10^−4^
**NONHSAT248790.1**	0.00	0.05	4.36 × 10^−2^	**ENSG00000218336**	**TENM3**	0.55	2.03	4.97 × 10^−2^
**MSTRG.48669.1**	0.37	1.81	9.30 × 10^−4^	**ENSG00000006638**	**TBXA2R**	0.19	0.06	5.62 × 10^−20^
**MSTRG.48669.1**	0.37	1.81	2.52 × 10^−4^	**ENSG00000179841**	**AKAP5**	3.28	1.56	4.97 × 10^−2^
**MSTRG.48669.1**	0.37	1.81	4.15 × 10^−2^	**ENSG00000169239**	**CA5B**	1.76	0.85	4.50 × 10^−10^
**NONHSAT252820.1**	0.77	0.10	6.29 × 10^−6^	**ENSG00000120278**	**PLEKHG1**	0.04	0.00	3.59 × 10^−28^
**NONHSAT176568.1**	12.50	0.05	1.77 × 10^−2^	**ENSG00000177885**	**GRB2**	17.81	6.81	8.86 × 10^−16^
**NONHSAT209182.1**	1.31	0.02	4.92 × 10^−4^	**ENSG00000244694**	**PTCHD4**	0.78	0.24	2.35 × 10^−14^
**ENST00000530595**	0.00	0.26	7.37 × 10^−3^	**ENSG00000166793**	**YPEL4**	0.06	0.01	1.29 × 10^−14^
**NONHSAT213922.1**	0.60	0.03	2.99 × 10^−2^	**ENSG00000234985**	**AC074085.2**	0.44	0.00	5.62 × 10^−20^

^a^ represents the expression of transcripts in the TiO_2_ NPs and control groups (FPKM).

## Data Availability

Not applicable.

## Supplementary Materials

The following supporting information can be downloaded at: https://www.mdpi.com/article/10.3390/ijerph20021059/s1, Figure S1: KEGG analysis of differentially expressed lncRNAs target genes.
