# Supplementary material for: Titanium Dioxide Nanoparticles Altered the lncRNA Expression Profile in Human Lung Cells
Source: Int J Environ Res Public Health. 2023 Jan 6;20(2):1059. doi: 10.3390/ijerph20021059 (PMC9858630; doi:10.3390/ijerph20021059)
Supplement: Supplementary file 1 [file ijerph-20-01059-s001.zip › ijerph-2088677-supplementary.pdf]

Supplementary Information

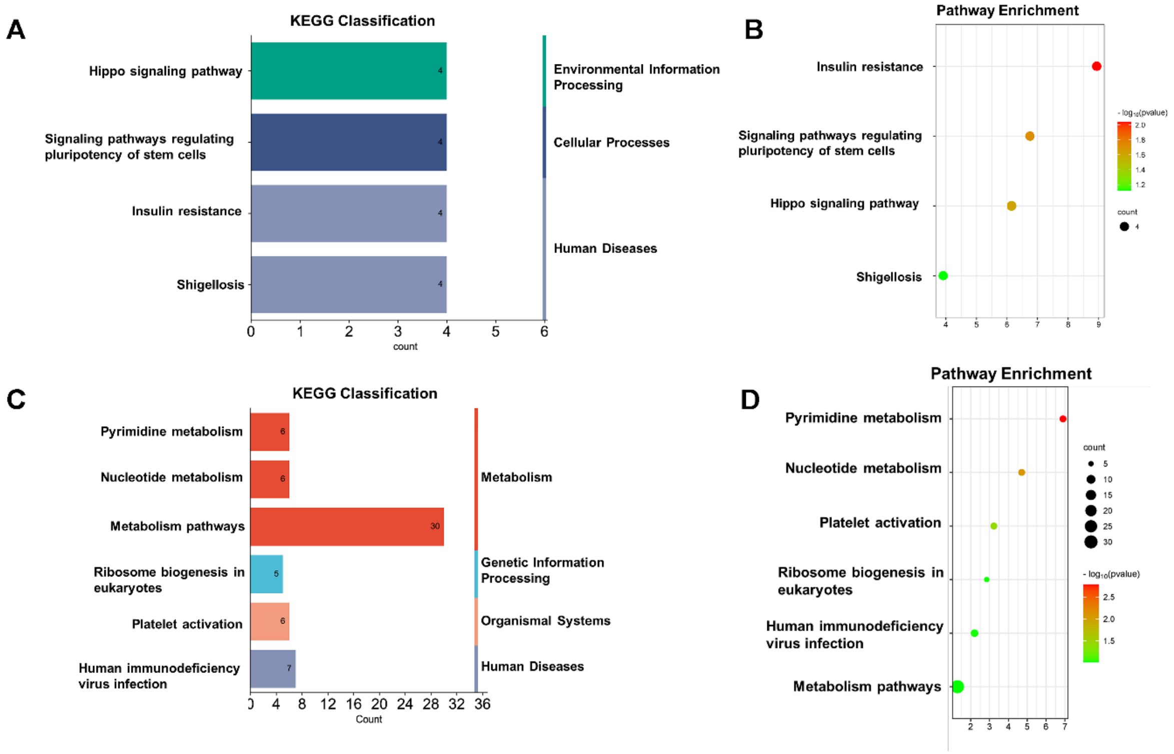

Figure S1. KEGG analysis of differentially expressed lncRNAs target genes. (A) The number of upregulated lncRNAs' target genes KEGG classifications was shown; (B) The results of KEGG pathway enrichment analysis of upregulated lncRNAs' target genes were shown in scatter plots; (C) The number of downregulated lncRNAs' target genes KEGG classifications was shown; (D) The results of KEGG pathway enrichment analysis of downregulated lncRNAs' target genes were shown in scatter plots.
